# Mistletoe as a keystone resource: an experimental test

**DOI:** 10.1098/rspb.2012.0856

**Published:** 2012-07-11

**Authors:** David M. Watson, Matthew Herring

**Affiliations:** Institute for Land, Water and Society, Charles Sturt University, PO Box 789, Albury, New South Wales 2640, Australia

**Keywords:** parasitic plant, removal experiment, eucalypt woodland, Loranthaceae, Santalales, facilitation

## Abstract

Various entities have been designated keystone resources, but few tests have been attempted and we are unaware of any experimental manipulations of purported keystone resources. Mistletoes (Loranthaceae) provide structural and nutritional resources within canopies, and their pervasive influence on diversity led to their designation as keystone resources. We quantified the effect of mistletoe on diversity with a woodland-scale experiment, comparing bird diversities before and after all mistletoe plants were removed from 17 treatment sites, with those of 11 control sites and 12 sites in which mistletoe was naturally absent. Three years after mistletoe removal, treatment woodlands lost, on average, 20.9 per cent of their total species richness, 26.5 per cent of woodland-dependent bird species and 34.8 per cent of their woodland-dependent residents, compared with moderate increases in control sites and no significant changes in mistletoe-free sites. Treatment sites lost greater proportions of birds recorded nesting in mistletoe, but changes in species recorded feeding on mistletoe did not differ from control sites. Having confirmed the status of mistletoe as a keystone resource, we suggest that nutrient enrichment via litter-fall is the main mechanism promoting species richness, driving small-scale heterogeneity in productivity and food availability for woodland animals. This explanation applies to other parasitic plants with high turnover of enriched leaves, and the community-scale influence of these plants is most apparent in low productivity systems.

## 1. Introduction

The idea of an ecological keystone was first transferred from an individual species to a generalized resource derived from multiple species by Leighton & Leighton [1] (see also Terborgh [2]) in their work on palm seeds, figs and nectar as food for birds and mammals in a Bornean forest. Since then, lichens, saguaro cacti, mineral licks, water, honeydew, mistletoes, salmon, acorns and various fruiting trees *inter alia* have been proposed to represent keystone resources, either within a particular region or generally [3–5]. With one notable exception [6], there have been no manipulations to quantify the direct and indirect influence of purported keystone resources. With most resources, removal would be logistically difficult, if not impossible, and various procedural difficulties confound the design or interpretation of supplementation experiments. Although our understanding of keystone species has been informed by numerous experiments (natural and controlled; removal and addition), the lack of manipulative tests represents a major impediment to advancing our understanding of how purported keystone resources influence diversity.

Engaged in a network of interdependencies with host plants, seed dispersers, pollinators and natural enemies, mistletoes are a group of highly interactive plants that have been proposed to represent keystone resources in forested ecosystems worldwide [7]. In addition to many regular and occasional consumers of their enriched foliage, abundant fruit and nectar resources offered when little else is available [8–10], mistletoe clumps are popular nest and roost sites [11–13] and numerous studies have reported positive relationships between mistletoe occurrence and species richness [10,14]. Mistletoe occurrence and density also affect several ecosystem processes: their abundant enriched litter has pronounced positive effects on nutrient dynamics and understorey composition [15,16] and changes to infected hosts increase colonization and functional diversity of mycorrhizal communities [17,18], increasing canopy complexity and changing fire behaviour and severity [19], thereby altering successional dynamics at the stand-scale [20].

Having called for removal experiments to test the keystone status of mistletoes, a series of predictions were outlined [7], detailing the short- and medium-term effects of mistletoe removal on community composition and structure. If mistletoe represents a keystone resource, areas where mistletoe has been removed would be expected to have:

1. lower abundances of mistletoe-obligate frugivores and folivores, with local populations declining towards local extinction;
2. lower abundances of regular mistletoe foragers (folivores, frugivores and nectarivores);
3. lower abundances of species that nest in mistletoe clumps and hollows;
4. lower richness of vertebrates generally; and
5. increased sensitivity to drought and other extreme events, supporting fewer residents and displaying increased seasonal and inter-annual variability in species richness, compared with control plots with typical numbers of mistletoe plants (after Watson [7]).

These predictions were provisionally tested by comparing two adjacent eucalypt woodlands, from one of which all mistletoe plants had been removed from one woodland five years prior to study [6]. Marked differences in avian species richness and incidence were detected, consistent with the predicted effects of mistletoe on resource availability, offering preliminary support for the keystone resource hypothesis. This study had an effective sample size of one and no pre-treatment data were collected, so the recorded effects may include pre-existing habitat differences and may not be representative of the influence of mistletoe more generally.

Here, we report on a large-scale experiment evaluating the influence of mistletoe on community-level diversity and designed to test these five predictions. Pre-treatment bird diversities (estimated at the woodland scale) were compared explicitly with diversities three years post-treatment, contrasting changes in woodlands where all mistletoes were removed with those in control woodlands where mistletoe numbers were unmodified. As the first experimental test of a purported keystone resource, to our knowledge this 6-year study yielded a refined understanding of the direct and indirect effects of mistletoe on diversity and community composition. More broadly, this study contributes to a growing understanding of the role of parasitic plants as facilitators, affecting occurrence patterns of other plants and dependent animals through altered nutrient dynamics.

## 2. Methods

### (a) Study area and site selection

This study was conducted in the upper Billabong Creek catchment in southeastern New South Wales, Australia [21] (for a map, see electronic supplementary material, figure S1), located at the transition between xeric plains to the west and mesic highlands to the east [22]. Elevation ranges between 220 to 889 m a.s.l., and average annual rainfall increases from approximately 550 mm in the west to 900 mm in the east of the catchment. The study period (2003–2008) coincided with a prolonged drought affecting southeastern Australia, with annual rainfall totals for the nearby Albury weather station being approximately half of the long-term annual average of 770 mm.

Rather than individual trees or stands, entire woodland remnants were used as study units, isolated 80–100 years ago as surrounding habitats were cleared for agriculture. Sites qualified for selection if: canopy cover was more than 5 per cent; fragment area was 1.5–25 ha (larger woodlands were not considered because complete mistletoe removal was deemed prohibitively difficult); vegetation was classed as either Dry Foothill Forest or Grassy Box Woodland; the woodland had ‘hard’ edges *sensu* [23] and was at least 500 m from the nearest study site. The ground layer of all woodlands was dominated by grasses (both native perennials, and native and exotic annuals) with occasional shrubs (*Acacia* spp., *Exocarpus* spp.) in low-to-very-low densities. Mistletoe plants occurred at medium-to-low densities in these woodlands—primarily *Amyema miquelii* but with occasional *Muellerina eucalyptoides*, *Am. pendula* and *Am. miraculosa* (Loranthaceae). Except for the latter species (principally epiparasitic on *Am. miquelii* in this part of its range), these species are primarily eucalypt parasites, the plants forming dense pendulous clumps at the edge of the canopy [24].

### (b) Bird surveys

To estimate bird species richness in the 40 woodlands, inventories were compiled from eight patch-scale surveys that were conducted each season in 2003/2004 prior to treatment and again in 2007/2008, three years after treatment. Surveys were conducted using the standardized search [25,26] with sample duration set at 20 min, using a quantitative, results-based stopping rule to determine the number of samples per survey. The stopping rule applied—stop sampling once observed richness of woodland-dependent species exceeds 80 per cent of predicted richness using the Chao 2 estimator [27]—yielded surveys of between three and six samples (i.e. 60–120 min of continuous sampling), with 260 of the 320 surveys tripping the stopping rule after three samples. Sampling involved walking throughout the woodland remnant and identifying all birds seen or heard within the woodland, including species flying beneath the canopy. Sampling was conducted only during favourable weather conditions, avoiding periods of heavy rain, strong wind or intense heat.

In addition to yielding richness estimates of uniform completeness, this approach generated incidence measures for each species in each woodland remnant, expressed as the proportion of samples in which it was detected [25]. Two incidence totals were calculated for each site in both years: summed incidence of all species that regularly feed on mistletoe nectar and/or fruit using the list compiled by Reid [28], and summed incidence of all species that nest in mistletoe clumps using [12]. In addition to total richness and richness of woodland-dependent species (after Watson [6] and references therein), a third richness measure was used for analysis—resident richness: those woodland-dependent species recorded in at least two seasons for the given year (excluding transients that were detected only in a single season). Analysis was restricted to changes in richness and incidence—interactions with patch and landscape-scale factors and comparisons of the individual responses of particular species and functional groups will be explored in subsequent contributions.

### (c) Experimental design

The woodland remnants selected for study varied considerably in management history, area and degree of isolation from other remnant vegetation, potentially confounding the effects of mistletoe on diversity patterns. To guard against this, we ensured treatment and control groups were comparable, assigned treatment (mistletoe removal) using a blind stratified random approach and compared the effects of treatment in terms of proportional rather than absolute change. To assign treatment, woodland area was plotted against percentage tree cover, yielding a scatter plot of 40 points distinguishing larger and more densely wooded remnants from smaller, more open woodlands. Points closest to one another in graphical space were paired and treatment assigned by coin toss, yielding two groups of 20 woodland fragments with similar ranges of tree density and patch area (electronic supplementary material, table S1). A series of tests confirmed that these two groups exhibited comparable mean values for woodland area, percentage of tree cover in the surrounding 1 km radius buffer (an index of habitat openness), area of tree cover in the surrounding 1 km radius buffer (a measure of land-use intensity), mistletoe abundance and species richness of woodland-dependent birds prior to treatment. Some woodlands did not contain mistletoe, so, rather than a simple binary comparison, there were three groups: control woodlands with mistletoe (*n* = 11), treatment patches with mistletoe plants removed (*n* = 17) and woodlands from which mistletoe was naturally absent (*n* = 12); hereafter, they are referred as ‘control’, ‘treatment’ and ‘mistletoe-free’ woodlands, respectively.

### (d) Mistletoe removal

Mistletoe plants growing within treatment woodlands were systematically removed over a five-month period (winter and spring of 2004) by teams of volunteers using pole-mounted loppers and pruning saws. Unlike some groups of mistletoe (e.g. *Arceuthobium* spp., Viscaceae) that cause systemic infections throughout the host, these loranthaceous mistletoes can be removed by cutting proximal to the haustorium, the lack of cortical strands in these species precluding re-sprouting [24]. Hydraulic boom-lifts towed by four-wheel drive vehicles were used to access plants up to 18 m above the ground, the telescoping boom arm allowing access to most parts of the eucalypt canopies (electronic supplementary material, figure S2). Mistletoe plants and associated sections of host branches were left where they fell: beneath their former hosts. Sham removals were conducted in the control sites, driving within the woodland and removing branches from infected and non-infected trees, but leaving all mistletoe plants intact. Tree and branch selection was haphazard, determined partly by ease of access and partly to avoid any mistletoe clumps within the canopy. Comprehensive surveys conducted six months and three years after initial treatment revealed that not all mistletoe plants were removed, but less than four per cent of the original number of mistletoe plants remained or recolonized, which were subsequently removed in follow-up treatment in the summer of 2006/2007.

### (e) Data manipulation and analysis

Responses to mistletoe removal were measured in terms of net change between pre- and post-treatment inventories. In some cases, this was simply the datum for the 2003/2004 subtracted from the equivalent datum for 2007/2008. To make these values more meaningful, many of these differences were expressed as proportions of the original (pre-treatment) value. The only mistletoe-obligate frugivore detected was the mistletoebird *Dicaeum hirundinaceum*, so prediction 1 was tested by comparing mistletoebird incidence before and after treatment. Predictions 2 and 3 were tested using summed incidence of mistletoe-feeding and mistletoe-nesting species (respectively), comparing control and treatment woodlands in terms of the difference in the net change of summed incidence. Tests were conducted on the actual values, while plots correspond to proportions of total incidence (pre-treatment) to place the differences in biological context. Prediction 4 was tested by comparing the change in species richness in control versus treatment woodlands, expressed as a proportion of the pre-treatment value (i.e. proportional change). For prediction 5, the study period coincided with a prolonged drought, with many species responding by leaving the study area completely or only occupying some habitats seasonally. The change in numbers of residents between the two years was expressed as a proportion of the initial value (i.e. the proportion decreasing as the number of transient species increased). Except for prediction 1, which entailed a paired *t-*test applied to the 17 treatment woodlands, all treatment effects were tested using Mann–Whitney–Wilcoxon tests (one-tailed) comparing mean values for the 17 treatment sites (with mistletoe plants removed) with the 11 control sites (with mistletoe left intact), with exact probabilities of less than 0.05 deemed significant (using spss v. 17.0). Tests were conducted using all species (‘total species’) and the subset of species considered to depend on woodland as their primary habitat (‘woodland species’; excluding most raptors, aerial foragers, open country and exotic species, after Watson [6]). Non-parametric tests were used, as sample sizes were uneven and the data were generally heteroscedastic, and one-tailed tests were appropriate as all comparisons were testing explicitly directional predictions [7]. In addition to comparing treatment and control sites, values for mistletoe-free woodlands are presented to contextualize the changes associated with experimental treatment and represent background variation between sites and years not associated with experimental manipulation.

## 3. Results

Mistletoe exhibited a highly irregular distribution across the 40 sites, being entirely absent from 12 sites and occurring in remaining sites at densities ranging from less than one per hectare to almost 200 per hectare. A total of 5493 mistletoe plants were removed: the great majority (5169; 94%) during the initial removal phase in mid-2004 and an additional 324 plants in follow-up removals in summer 2006/2007, most of which were immature plants.

Of the 75 woodland-dependent bird species recorded in the two years combined, 12 species were recorded only during pre-treatment surveys and two species were recorded only during post-treatment surveys—i.e. there was a net loss of 10 species, or 14 per cent of the pre-treatment diversity of woodland-dependent species across all 40 sites. Turnover in the other 41 species was more symmetrical, with seven species recorded only pre-treatment (2003/2004) and eight species recorded only post-treatment (2007/2008)—i.e. a net gain of one species (or 3% of the pre-treatment total). Additional detail on bird richness and mistletoe occurrence is summarized in the electronic supplementary material.

### (a) Prediction 1: specialist frugivores

A significant difference in mistletoebird *D. hirundinaceum* incidence was detected in the 17 treatment sites (*t* = 1.825, *p* = 0.043, one-tailed), reflecting declines in the treatment woodlands following mistletoe removal—incidence decreased in two sites and the species became locally extinct in a further four. By contrast, mistletoebird incidence increased in five of the 11 control woodlands, decreased in three others and they were not recorded in the remaining three.

### (b) Prediction 2: mistletoe foragers

**Figure 1.**
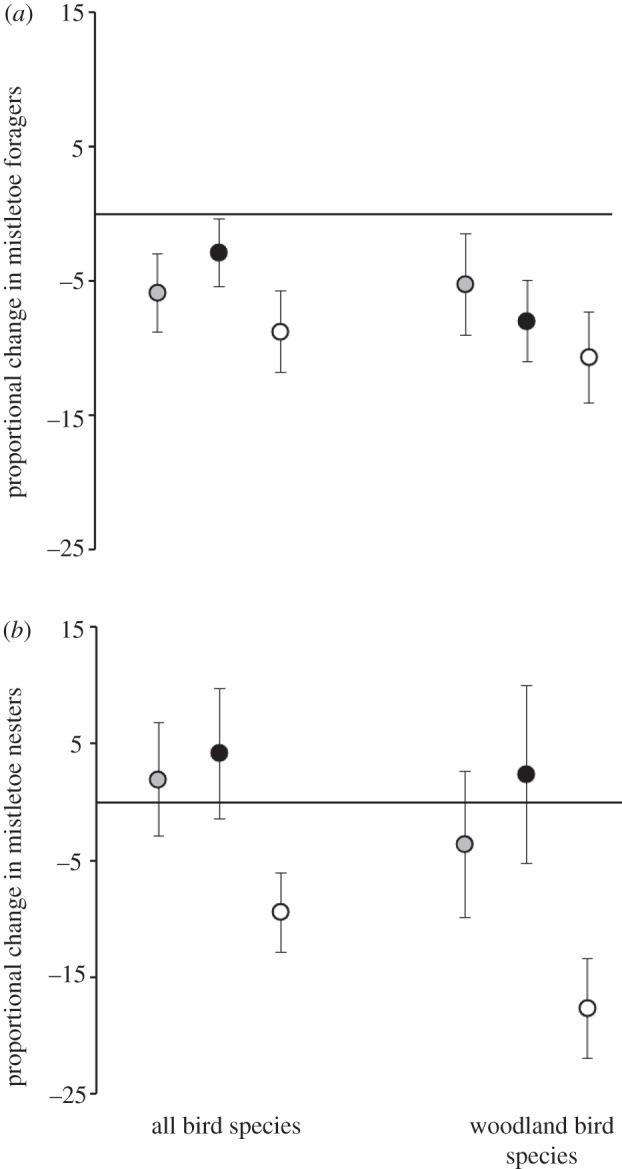
Proportional change in summed incidence of bird species between the two years (2003/2004 and 2007/2008) were compared using Mann–Whitney–Wilcoxon comparison of means (one-tailed), for the 12 mistletoe-free sites (grey), 11 control sites (black) and 17 treatment sites (white). (*a*) Summary of changes for those species that regularly forage on mistletoe nectar and/or fruit (31 species, of which 24 are woodland-dependent; after Reid [28]). No significant differences were detected, and prediction 2 was not supported. (*b*) Summary of changes for those species that have been recorded nesting in mistletoe clumps (67 species, of which 48 are woodland-dependent; after Cooney *et al*. [12]). Significant differences between means for treatment and control sites were detected (*p* = 0.008); treatment sites lost a greater proportion of mistletoe nesters than control sites, supporting prediction 3.

No significant differences were detected between treatment and control sites in terms of net change in summed incidence of all 31 mistletoe foragers (*p* = 0.132, one-tailed), or for the 24 woodland-dependent mistletoe foragers (*p* = 0.373, one-tailed) for ranked data (figure 1*a*) [28,12]. There were decreases across all sites, with treatment sites losing an average of 8.7 per cent of summed incidence for total species and 10.6 per cent of woodland-dependent species (figure 1*a*), and control sites losing 2.9 per cent and 8 per cent, respectively. Note that these recorded losses were less than changes in summed incidence across all species in the treatment woodlands between years (decreases of 15.4% and 24.7%, respectively).

### (c) Prediction 3: mistletoe nesters

Clear differences in summed incidence of the 67 mistletoe nesters were detected between treatment and control woodlands (*p* = 0.016, one-tailed), equating to a mean loss of approximately 9.4 per cent of total incidence (s.e. = 3.4) in treatment woodlands, becoming more marked if just the 48 woodland-dependent species were considered (*p* = 0.008, one-tailed; mean loss of 17.6% of total incidence, s.e. = 4.26). Although significantly greater than decreases in control woodlands (figure 1*b*), these changes were less than change in incidence across all species in the treatment sites (decreases of 15.4% for all species; 24.7% for woodland-dependents).

### (d) Prediction 4: species richness

**Figure 2.**
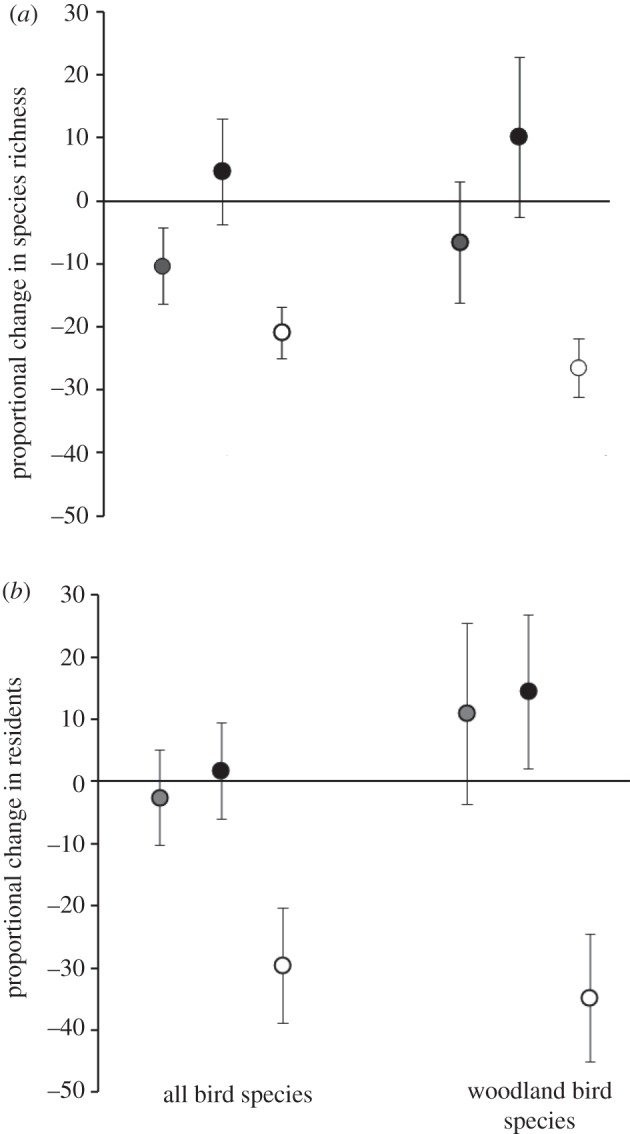
(*a*) Proportional change in species richness between 2003/2004 and 2007/2008 was compared using Mann–Whitney–Wilcoxon comparison of means (one-tailed), for the 12 mistletoe-free sites (grey), 11 control sites (black) and 17 treatment sites (white). Treatment sites lost significantly more species than control sites, in terms of both total species (*p* = 0.004) and woodland-dependent species (*p* = 0.002), lending support to prediction 4. (*b*) The effect of mistletoe removal on sensitivity to drought was tested by comparing the proportional change in residents after mistletoe removal. Treatment sites lost significantly more residents than control sites (*p* = 0.008 for total residents; *p* = 0.002 for woodland-dependent residents), providing support for prediction 5.

Total species richness decreased markedly in the treatment woodlands after experimental removal of mistletoe—a significant difference compared with control woodlands (*p* = 0.004), reflecting mean losses of 20.9 per cent of their original richness (s.e. = 4.04). This pattern was more marked for those species dependent on woodlands as their principal habitat (*p* = 0.002, one-tailed; compared with control sites), equating to mean losses of 26.5 per cent (s.e. = 4.58) of their original woodland-dependent species richness (figure 2*a*). This change compares with a mean decrease in total richness of 10.3% (s.e. = 6.04) in mistletoe-free woodlands and an increase of 4.7 per cent (s.e. = 8.38) in the control woodlands (for total species richness); with woodland-dependent species richness decreasing by an average of 6.5 per cent in mistletoe-free woodlands (s.e. = 9.63), while control woodlands gained an average of 10.2 per cent of their pre-treatment richness (s.e. = 12.68).

### (e) Prediction 5: sensitivity to drought

For total species, clear-cut differences between control and treatment were detected (*p* = 0.008): treatment sites lost a mean proportion of 29.5 per cent (s.e. = 9.25) of their initial residents, while control sites exhibited an increase in the proportion of residents of 1.73 per cent (s.e. = 7.73). Differences in woodland-dependent species were greater (*p* = 0.002); control woodlands increased their proportion of residents (14.5%; s.e. = 12.34) while the proportion of residents in treatment woodlands decreased by 34.8 per cent (s.e. = 10.3), transient species becoming more dominant (figure 2*b*).

## 4. Discussion

The influence of mistletoe on diversity was evaluated directly with a woodland-scale removal experiment, and the hypothesized status of these hemiparasites as keystone resources was strongly supported. Removing mistletoe plants from entire woodlands resulted in average losses of more than a quarter of the woodland-dependent bird species, with the number of resident species decreasing by more than a third. Over the same period, control woodlands with variable mistletoe densities exhibited moderate increases in woodland-dependent species richness, while no marked changes to richness or incidence were detected in naturally mistletoe-free sites. Rather than affecting just those species that feed on mistletoe or nest preferentially in mistletoe clumps, these changes were apparent across the community, being more pronounced for woodland-dependent species and residents. The magnitude of these changes within three years of mistletoe removal provides clear evidence that mistletoes directly modify resource availability in woodlands and forests, consistent with previous descriptive work relating mistletoe density to community-level diversity in a range of other ecosystems [10,14].

Two aspects of this study probably underestimate the measured influence of mistletoe, so reported effect sizes should be regarded as conservative. The study period coincided with the final years of a prolonged drought affecting southern Australia, so the pre-treatment inventories yielded lower species richness estimates than anticipated. Rather than supporting comparable bird diversities in the two years, the 11 control woodlands gained an average of 10.2 per cent woodland-dependent species because numbers of drought-sensitive species rebounded in 2007. So the mean-recorded loss of 26.5 per cent woodland-dependent species in the treatment remnants may actually reflect net losses closer to 36.7 per cent species richness once the confounding effects of regional climatic conditions are removed, comparable to the 34.8 per cent mean-recorded loss of woodland-dependent residents. Second, mistletoe densities within the study sites were low compared with other habitats in the region, the hemiparasites being completely absent from 12 of the 40 woodlands. The same mistletoe species are two to five times more abundant in adjacent riparian and floodplain woodlands and nearby box-ironbark forests, probably having more pronounced effects on diversity and community composition.

### (a) Mistletoe as a direct nutritional resource

One of the main elements of the evidence base assembled to justify designation of mistletoe as a keystone resource [7] was dietary information for terrestrial vertebrates, with the popularity of mistletoe fruit, nectar and foliage deemed instrumental in explaining observed relationships with species richness. Although mistletoe specialists declined following mistletoe removal (in support of prediction 1), regular mistletoe foragers did not show significant reductions in occurrence or abundance following mistletoe removal (figure 2*a*). While it is likely that more of the bird species recorded in this study occasionally consume mistletoe fruit or nectar, Reid's [28] list corresponds closely with our own observations of nectarivory and frugivory, both within the study region and across southern Australia, and this negative result is considered robust.

We suggest that regular consumers of mistletoe fruit and nectar represent only a small component of the suite of species influenced by mistletoe occurrence in this system, and that direct nutritional effects comprise a relatively minor component of the community-level influence of mistletoe in general. Eucalypt forests are remarkable for their lack of fleshy fruited plants, and frugivorous species are only a minor component of the associated avifauna. Thus, changes in mistletoe diversity and abundance probably have more pronounced effects on frugivore occurrence in other systems, explaining temporal and spatial variation in frugivores numbers [8,29]. In terms of nectar, although 13 of the 75 recorded woodland-dependent species feed on mistletoe flowers and an additional seven visit mistletoe flowers opportunistically [28], none depends on mistletoes as their primary food source. Rather, eucalypts represent the dominant nectar-bearing plant in this region—the canopy-dominant sclerophyllous trees exhibiting mass flowering events that are highly variable through space and time, driving large-scale movements as nectarivores track peak flowering across large distances [30]. Hence, the direct influence of mistletoe fruit and nectar availability on community-scale diversity may not be as consistently great as predicted, and the influence of more abundant fruit and nectar-bearing plants in the canopy or understorey is probably far greater in most systems [31,32].

### (b) Structural attributes

In addition to nutritional resources, mistletoes provide dense evergreen structures within forest canopies that are widely used by birds and mammals for nesting and roosting, and these structural attributes formed the second main justification for designating mistletoes as keystone resources. Accordingly, those species that use mistletoe for nest sites were expected to decrease in abundance following mistletoe removal (prediction 3). Although treatment sites displayed significant reductions in these species in terms of summed incidence, recorded changes were less than those exhibited across all woodland-dependent species—i.e. mistletoe-nesting species were no more likely to undergo declines following mistletoe removal than any other woodland species. This conclusion is supported by the relatively small number of nests found during mistletoe removal—fewer than one per cent of the plants removed had nests associated with them. So, although widely used as nesting and roosting sites, and preferentially selected by some species, we found no evidence that mistletoes were limiting as a nesting substrate, nor that woodlands with mistletoe can support greater abundances of mistletoe-nesting species.

Previous research has evaluated mistletoe nesting in more depth, and suggested that microclimatic factors may be important in explaining the widespread practice. Compared with nests elsewhere in the canopy, nests within mistletoe foliage experienced reduced fluctuation in both temperature and humidity, with the ambient climate ameliorated by the semi-succulent foliage of the hemiparasite [13]. The lack of signal detected here may reflect the temperate climate of the study region, and mistletoe is likely to have more pronounced effects in areas with more extreme climates. Indeed, research on wildlife use of dwarf mistletoe brooms in Arizona found evidence that mistletoes may be a limiting resource [33]. The influence of mistletoe on canopy structure may be better considered in terms of reproductive success rather than in terms of species richness, increasing the seasonal extent and geographical breadth within which successful fledging and recruitment can be achieved.

### (c) Facilitation via litter-fall and nutrient concentration

Rather than direct nutritional supplementation or increased structural complexity in infected host canopies, we propose that the marked influence of mistletoe on diversity is mediated primarily via the abundant enriched litter shed by these hemiparasites, increasing productivity and promoting coexistence through bottom-up trophic dynamics of woodland and forest food webs. Previous work on the contribution of mistletoes to litter-fall and nutrient dynamics within the study area estimated that individual *Amyema miquelii* plants contributed an average of 544 g of leaf litter annually, with 0.81 ± 0.08 g of mistletoe litter produced per gram of mistletoe leaf biomass in the canopy [15]. The variation in mistletoe occurrence noted at the patch scale was even more pronounced within infected patches: mistletoe plants aggregated in discrete ‘infection centres’ and concentrated around the perimeter of remnants. Hence, rather than incremental changes in litter-fall across woodlands, mistletoe occurrence increases heterogeneity in litter-fall, effectively doubling or tripling total litter-fall in heavily infected stands. The effects of these qualitative changes in litter-fall are magnified by the enriched status of mistletoe litter compared with host litter, leading to marked increases in nutrient inputs [16] and pronounced spatial heterogeneity in nutrient returns.

In addition to affecting overall productivity and composition of understorey plant communities [15], these changes to the litter layer directly affect the litter-dwelling arthropods that form the principal food source for many woodland-dependent fauna. Previous work on the habitat preferences, dietary composition and foraging ecology of birds in eucalypt woodlands has consistently identified litter depth as a critical factor [34 and references therein], with deeper and more extensive litter beds considered to contain higher abundances of preferred prey [35]. Thus, we suggest that quantitative changes to litter-fall associated with mistletoe occurrence, plus qualitative changes in the chemical composition and associated rate of decomposition of resultant litter, result in fundamental changes to litter-dwelling arthropod communities, increasing and prolonging availability of prey for insectivores.

Rather than supplanting direct nutritional and structural factors, the facilitation explanation incorporates these pathways, with nutrient contributions by foraging and nesting animals adding to the inputs provided directly by mistletoes. Unlike litter-fall that represents reallocation of nutrients from the host to the litter-layer beneath the host, these animal-derived nutrients are drawn from a much larger pool, extending beyond the infected host and stand [36]. Similar mechanisms have been postulated to explain high concentrations of animal-derived elements near hollow trees, rock outcrops and copses of trees surrounded by grassland, summarized by Watson [37]. Rather than simply increased productivity, we suggest it is the heterogeneity of nutrient availability driven by the highly aggregated distribution of mistletoes that underpins their pervasive effects on diversity, boosting species richness by promoting species coexistence.

### (d) Effects of parasitic plants on diversity

Rather than being unique to this system, nutrient enrichment is an attribute of parasitic plants generally, and numerous studies (of other mistletoes and various root parasites) have reported findings consistent with facilitation via enriched litter-fall. These findings involve a range of vegetation types, from alpine tundra [38] and arctic shrubland [39] to coniferous forests [40]. In addition to direct effects of hemiparasite litter on decomposition and nutrient availability, these studies demonstrate various indirect effects of parasitic plants, including the boosting of species richness by preferentially parasitizing competitively dominant species [41,42]. Parallel research on dwarf mistletoes (Arceuthobium: Viscaceae) has detected increased colonization of infected *Pinus edulis* by ectomycorrhizae [17] and increased functional diversity of fungal communities beneath infected *Pinus contorta* [18], suggesting that the fundamental shifts in soil microbial communities detected by Bardgett *et al*. [43] in grasslands experimentally infected with *Rhinanthus minor* may apply to parasitic plants more generally.

## References

[1] Leighton M. Leighton D. R. 1983 Vertebrate responses to fruiting seasonality within a Bornean rain forest. In Tropical rainforest: ecology and management (eds Sutton S. L. Whitmore T. C. Chadwick A. C.), pp. 181–196 Cambridge, UK: Blackwell Scientific Publications

[2] Terborgh J. 1983 Five new world primates: a study in comparative ecology. Princeton, NJ: Princeton University Press

[3] Terborgh J. 1986 Keystone plant resources in the tropical forest. In Conservation biology: the science of scarcity and diversity (ed. Soulé M.), pp. 330–344 Sunderland, MA: Sinauer

[4] Shachak M. Jones C. G. Granot Y. 1987 Herbivory in rocks and the weathering of a desert. Science 236, 1098–1099 10.1126/science.236.4805.1098 (doi:10.1126/science.236.4805.1098) 17799665

[5] Power M. E. 1996 Challenges in the quest for keystones. BioScience 46, 609–620 10.2307/1312990 (doi:10.2307/1312990)

[6] Watson D. M. 2002 Effects of mistletoe on diversity: a case-study from southern New South Wales. Emu 102, 275–281 10.1071/MU01042 (doi:10.1071/MU01042)

[7] Watson D. M. 2001 Mistletoe: a keystone resource in forests and woodlands worldwide. Ann. Rev. Ecol. Syst. 32, 219–249 10.1146/annurev.ecolsys.32.081501.114024 (doi:10.1146/annurev.ecolsys.32.081501.114024)

[8] Irwin M. T. 2008 Feeding ecology of diademed sifakas (*Propithecus diadema*) in forest fragments and continuous forest. Int. J. Primatol. 29, 95–115 10.1007/s10764-007-9222-9 (doi:10.1007/s10764-007-9222-9)

[9] Bowen M. E. McAlpine C. A. House A. P. N. Smith G. C. 2009 Agricultural landscape modification increases the abundance of an important food resource: mistletoes, birds and brigalow. Biol. Cons. 142, 122–133 10.1016/j.biocon.2008.10.005 (doi:10.1016/j.biocon.2008.10.005)

[10] Montague-Drake R. M. Lindenmayer D. B. Cunningham R. B. 2009 Factors affecting site occupancy by woodland bird species of conservation concern. Biol. Cons. 142, 2896–2903 10.1016/j.biocon.2009.07.009 (doi:10.1016/j.biocon.2009.07.009)

[11] Cooney S. J. N. Watson D. M. 2005 Diamond firetails *Stagonopleura guttata* preferentially nest in mistletoe. Emu 105, 317–322 10.1071/MU05030 (doi:10.1071/MU05030)

[12] Cooney S. J. N. Watson D. M. Young J. 2006 Mistletoe as a nest site for Australian birds: a review. Emu 106, 1–12 10.1071/MU04018 (doi:10.1071/MU04018)

[13] Cooney S. J. N. Watson D. M. 2008 An experimental approach to understanding the use of mistletoe as a nest substrate for birds: nest predation. Wildl. Res. 35, 65–71 10.1071/WR06144 (doi:10.1071/WR06144)

[14] Bennetts R. E. White G. C. Hawksworth F. G. Severs S. E. 1996 The influence of dwarf mistletoe on bird communities in Colorado ponderosa pine forests. Ecol. Appl. 6, 899–909 10.2307/2269493 (doi:10.2307/2269493)

[15] March W. A. Watson D. M. 2007 Parasites boost productivity: effects of mistletoe on litterfall dynamics in a temperate Australian forest. Oecol. 154, 339–347 10.1007/s00442-007-0835-7 (doi:10.1007/s00442-007-0835-7) 17713788

[16] March W. A. Watson D. M. 2010 The contribution of mistletoes to nutrient returns in a temperate eucalypt forest: evidence for a critical role in nutrient cycling. Austral. Ecol. 35, 713–721 10.1111/j.1442-9993.2009.02056.x (doi:10.1111/j.1442-9993.2009.02056.x)

[17] Mueller R. C. Gehring C. A. 2006 Interactions between an above-ground plant parasite and below-ground ectomycorrhizal fungal communities on pinyon pine. J. Ecol. 94, 276–284 10.1111/j.1365-2745.2006.01105.x (doi:10.1111/j.1365-2745.2006.01105.x)

[18] Cullings K. Hanely J. 2010 Dwarf mistletoe effects on soil basidiomycete community structure, soil fungal functional diversity, and soil enzyme function: implications for climate change. Soil Biol. Biochem. 42, 1976–1981 10.1016/j.soilbio.2010.07.018 (doi:10.1016/j.soilbio.2010.07.018)

[19] Mathiasen R. L. Nickrent D. Shaw D. C. Watson D. M. 2008 Mistletoes: their pathological effects, molecular systematics, ecological importance, and management. Plant Dis. 93, 988–1002 10.1094/PDIS-92-7-0988 (doi:10.1094/PDIS-92-7-0988) 30769529

[20] Shaw D. C. Watson D. M. Mathiasen R. L. 2004 Comparison of dwarf mistletoes (*Arceuthobium* spp., Viscaceae) in the western United States with mistletoes (*Amyema* spp., Loranthaceae) in Australia: ecological analogs and reciprocal models for ecosystem management. Aust. J. Bot. 52, 481–498 10.1071/BT03074 (doi:10.1071/BT03074)

[21] Thackway R. Creswell I. D. 1995 An interim biogeographic regionalisation for Australia: a framework for establishing the national system of reserves. *v. 4.0* Canberra: Australian Nature Conservation Agency

[22] Caughley J. Gall B. 1985 Relevance of zoogeographical transition to conservation of fauna: amphibians and reptiles in the southwestern slopes of New South Wales. Aust. Zool. 21, 513–529

[23] Lindenmayer D. B. Fischer J. 2006 Habitat fragmentation and landscape change: an ecological and conservation synthesis. Washington, DC: Island Press

[24] Watson D. M. 2011 Mistletoes of southern Australia. Collingwood, Australia: CSIRO Publishing

[25] Watson D. M. 2003 The ‘standardized search’: an improved way to conduct bird surveys. Austral. Ecol. 28, 515–525 10.1046/j.1442-9993.2003.01308.x (doi:10.1046/j.1442-9993.2003.01308.x)

[26] Watson D. M. 2004 Comparative evaluation of new approaches to survey birds. Wildl. Res. 31, 1–11 10.1071/WR03022 (doi:10.1071/WR03022)

[27] Chao A. 1987 Estimating the population size for capture–recapture data with unequal catchability. Biometrics 43, 783–791 10.2307/2531532 (doi:10.2307/2531532) 3427163

[28] Reid N. 1986 Pollination and seed dispersal of mistletoes (Loranthaceae) by birds in southern Australia. In The dynamic partnership: birds and plants in southern Australia (eds Ford H. A. Paton D. C.), pp. 64–84 South Australia: Government Printer

[29] Barea L. Watson D. M. 2007 Temporal variation in food resources determines onset of breeding in an Australian mistletoe specialist. Emu 107, 203–209 10.1071/MU07003 (doi:10.1071/MU07003)

[30] Woinarski J. C. Z. Connors G. Franklin D. C. 2000 Thinking honeyeater: nectar maps for the Northern Territory, Australia. Pac. Cons. Biol. 6, 61–80

[31] Moegenburg S. M. Levey D. J. 2003 Do frugivores respond to fruit harvest? An experimental study of short-term responses. Ecology 84, 2600–2612 10.1890/02-0063 (doi:10.1890/02-0063)

[32] Kessler-Rios M. M. Kattan G. H. 2012 Fruits of Melastomataceae: phenology in Andean forest and role as a food resource for birds. J. Trop. Ecol. 28, 11–21 10.1017/S0266467411000642 (doi:10.1017/S0266467411000642)

[33] Hedwall S. J. Mathiasen R. L. 2006 Wildlife use of Douglas–Fir dwarf mistletoe witches’ brooms in the Southwest. West. N. Am. Nat. 66, 450–455 10.3398/1527-0904(2006)66[450:WUODDM]2.0.CO;2 (doi:10.3398/1527-0904(2006)66[450:WUODDM]2.0.CO;2)

[34] Watson D. M. 2011 A productivity-based explanation for woodland bird declines: poorer soils yield less food. Emu 111, 10–18 10.1071/MU09109 (doi:10.1071/MU09109)

[35] Razeng E. Watson D. M. 2012 What do declining woodland birds eat? A synthesis of dietary records. Emu 112, 149–159 10.1071/MU11099 (doi:10.1071/MU11099)

[36] Rawsthorne J. Watson D. M. Roshier D. A. 2011 Implications of movement patterns of a dietary generalist for mistletoe seed dispersal. Austral. Ecol. 36, 650–655 10.1111/j.1442-9993.2010.02200.x (doi:10.1111/j.1442-9993.2010.02200.x)

[37] Watson D. M. 2009 Parasitic plants as facilitators: more Dryad than Dracula? J. Ecol. 97, 1151–1159 10.1111/j.1365-2745.2009.01576.x (doi:10.1111/j.1365-2745.2009.01576.x)

[38] Spasojevic M. J. Suding K. N. 2011 Contrasting effects of hemiparasites on ecosystem processes: can positive litter effects offset the negative effects of parasitism? Oecologia 165, 193–200 10.1007/s00442-010-1726-x (doi:10.1007/s00442-010-1726-x) 20658151 PMC3015203

[39] Quested H. M. Press M. C. Callaghan T. V. 2003 Litter of the hemiparasite *Bartsia alpina* enhances plant growth: evidence for a functional role in nutrient cycling. Oecologia 135, 606–614 10.1007/s00442-003-1225-4 (doi:10.1007/s00442-003-1225-4) 12684861

[40] Stanton S. Hadley K. S. 2010 The influence of western dwarf mistletoe on surface fuels and snag abundance in mature ponderosa pine and mixed conifer stands in central Oregon. Nat. Areas J. 30, 261–270 10.3375/043.030.0302 (doi:10.3375/043.030.0302)

[41] Cameron D. D. Hwangbo J.-K. Keith A. M. Geniez J.-M. Kraushaar D. Rowentree J. Seel W. E. 2005 Interactions between the hemiparasitic angiosperm *Rhinanthus minor* and its hosts: from the cell to the ecosystem. Folia Geobot. 40, 217–229 10.1007/BF02803236 (doi:10.1007/BF02803236)

[42] Ameloot E. Verlinden G. Boeckx P. Verheyen K. Hermy M. 2008 Impact of hemiparasitic *Rhinanthus angustifolius* and *R. minor* on nitrogen availability in grasslands. Plant Soil 311, 255–268 10.1007/s11104-008-9640-2 (doi:10.1007/s11104-008-9640-2)

[43] Bardgett R. D. Smith R. S. Shiel R. S. Peacock S. Simkin J. M. Quirk H. Hobbs P. J. 2006 Parasitic plants indirectly regulate below-ground properties in grassland ecosystems. Nature 439, 969–972 10.1038/nature04197 (doi:10.1038/nature04197) 16495998

